# Role of Postbiotics in Diet-Induced Metabolic Disorders

**DOI:** 10.3390/nu14183701

**Published:** 2022-09-07

**Authors:** Miri Park, Minji Joung, Jae-Ho Park, Sang Keun Ha, Ho-Young Park

**Affiliations:** Food Functionality Research Division, Korea Food Research Institute, Wanju 55365, Korea

**Keywords:** postbiotic, metabolite, parabiotic, metabolic disorder, gut microbiota

## Abstract

Although the prevalence of metabolic disorders has progressively increased over the past few decades, metabolic disorders can only be effectively treated with calorie restriction and improved physical activity. Recent research has focused on altering the gut microbiome using prebiotics, probiotics, and postbiotics because various metabolic syndromes are caused by gut microbial dysbiosis. Postbiotics, substances produced or released by microorganism metabolic activities, play an important role in maintaining and restoring host health. Because postbiotics have a small amount of literature on their consumption, there is a need for more experiments on short- and long-term intake. This review discusses current postbiotic research, categories of postbiotics, positive roles in metabolic syndromes, and potential therapeutic applications. It covers postbiotic pleiotropic benefits, such as anti-obesity, anti-diabetic, and anti-hypertensive qualities, that could aid in the management of metabolic disorders. Postbiotics are promising tools for developing health benefits and therapeutic goals owing to their clinical, technical, and economic properties. Postbiotic use is attractive for altering the microbiota; however, further studies are needed to determine efficacy and safety.

## 1. Introduction

Metabolic syndrome is a broad cluster of symptoms that increases the risk of developing vascular and neurological complications such as cardiovascular disease, insulin resistance, diabetes, and cerebrovascular accidents [1]. Various metabolic diseases are related to each other, and individuals with metabolic syndrome often have three or more of the following conditions: obesity, hyperglycemia, hypertension, hypertriglyceridemia, and low high-density lipoprotein (HDL) cholesterol. Treatment of metabolic syndrome mainly includes lifestyle changes, diet, increased physical activity, and targeted drug therapy for specific affections. Currently, there are no single etiological factors or pivotal pathophysiological changes to effectively control disease progression. Current studies on the relationship between diet and metabolic syndrome focus on the efficacy of regulation of intestinal microflora in terms of weight maintenance and insulin sensitivity recovery, as well as calorie restriction and macronutrient supplementation.

The interaction between commensal bacteria and host cells in a healthy gut promotes the development of intestinal barrier function and immunological responses, both of which help to maintain host homeostasis [2,3]. In addition, much evidence indicates that an imbalance in the composition of the gastrointestinal microbial composition may contribute to obesity-related insulin resistance development as well as metabolic disorders such as hypertension and colitis [4,5,6,7]. This hypothesizes that modulating the gut microbiota may promote weight loss or prevent obesity in humans. However, it is unclear how and why the gut microbiome of obese hosts influences lipid metabolism to extract more energy from food. Bile acid metabolism, short-chain fatty acid (SCFA) production, and metabolic endotoxemia may be major causes of increased energy absorption by the gut microbiota [8]. This pathological condition further leads to low-grade systemic inflammation that can contribute to the pathogenesis of insulin resistance, diabetes, and obesity. Thus, the composition of the gut microbiota has the potential to influence the pathogenesis of several metabolic disorders.

Recently, “postbiotics” have gained popularity because they prevent metabolic syndrome by causing beneficial effects in intestinal microbiota. We summarized recent studies to comprehensively demonstrate the effectiveness of the administration of individual types of postbiotics in the prevention, alleviation, and treatment of diet-induced metabolic disorders due to the numerous beneficial effects of postbiotics and the concerns associated with the supplementation of live probiotic microbes.

## 2. Postbiotic Definition and Characteristics

**Figure 1:**
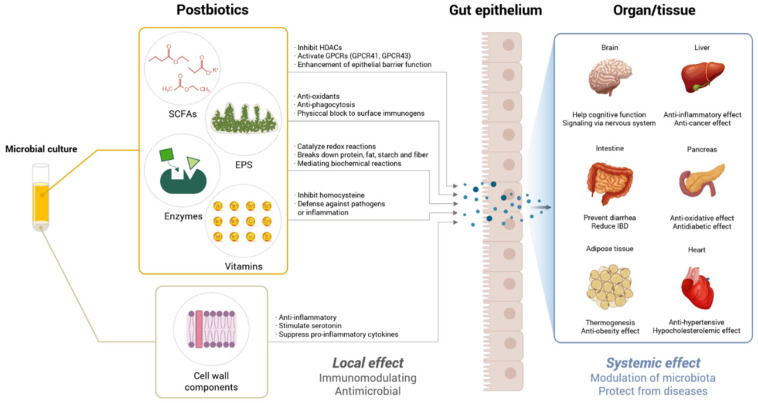
Schematic representation of various health benefits of postbiotic molecules in the host. EPSs, extracellular polymeric substances; GPCRs, G-protein-coupled receptors; HDACs, Histone deacetylases; SCFAs, short-chain fatty acids.

Postbiotics are metabolites produced after the beneficial gut bacteria metabolize prebiotics or probiotic components, or substances derived from the bacterial cell wall after cell wall destruction (Figure 1) [9,10]. The most commonly used approach for extracting postbiotics from microorganisms is to isolate metabolites produced by microbes using heat, enzymes, ultrasonication, ultraviolet treatment, centrifugation, and ultrafiltration procedures [11]. Fermentation using a proteolytic broth is also a widely utilized approach in the laboratory; postbiotic yield is increased when the pH is close to neutral [12].

The notion that destroyed microbes enhance or sustain health is not novel and several terms have been used to characterize them, including “parabiotics,” “ghost probiotics,” “inactivated probiotics,” “non-viable probiotics,” “heat-killed probiotics,” and “Tyndallized probiotics.” Parabiotics, also known as beneficial inactivated probiotics, refer to intact or ruptured microbial cells containing cellular components such as peptidoglycan, teichoic acid, and surface proteins, or crude cell extracts with complex chemical composition [13]. Postbiotics are complex mixtures of metabolites secreted by probiotics such as enzymes, secreted proteins, short-chain fatty acids, vitamins, secreted biosurfactants, amino acids, peptides, and organic acids from cell-free supernatants [14]. However, parabiotics have been used to refer to the entire category of postbiotics since they are known as postbiotics when inactive microbial components have biological activity in the host; similarly, “postbiotics” is used to refer to both terms here.

What is known as the probiotic effect is most likely a postbiotic effect. Interestingly, postbiotics overcome probiotic safety limitations [13]. Moreover, postbiotics have little interaction with food ingredients, are easy to process, and are advantageous for storage and shipping [15]. The International Scientific Association for Probiotics and Prebiotics defines probiotics as “living microorganisms that, when administered in appropriate doses, confer a health benefit on the host” [16]. However, it is difficult to maintain probiotic stability because these microorganisms must reach the host intestine without being killed by processing, storage, or passage through the gastrointestinal tract. Moreover, concerns have been raised about the functionality and safety of probiotics in immunocompromised people and the elderly [17]. In contrast, postbiotics easily deliver active ingredients to the intestine and may be used in places where storage and transport are difficult to maintain, such as in developing countries [14]. Postbiotics may be used as a substitute for people who have difficulty taking probiotics because of safety concerns [18]. Wegh et al. [14] report that postbiotics boost the effectiveness of active microorganisms or convert them into functional ingredients.

However, there are reports of limitations and potential side effects in the use of specific postbiotics for treating and preventing diseases. Three out of seven randomized comparative studies in children under the age of five found that postbiotics cause vomiting, bloating, and severe dehydration [19]. Thus, while postbiotics have several advantages over probiotics, additional studies are necessary to determine the biological effects and safeties.

## 3. Postbiotic Types and Features

Postbiotics include numerous compounds, most of which are secreted by microbes, such as SCFAs, exopolysaccharides (EPSs), enzymes, and vitamins [20,21,22]. Postbiotics also include chemicals that comprise cell structures, such as components of microbial cell walls and bacterial cultures [22]. As delineated in Table 1, postbiotics also possess anti-inflammatory, immunomodulatory, anti-obesity, and cholesterol-lowering properties. Postbiotic metabolites (e.g., SCFAs, EPSs, enzymes, and vitamins) produced by gut microbes are considered a direct measure to correct dysregulation of the gut microbiota and restore a healthy balance, independent of production by the microbiota. Metabolites that are administered directly as “postbiotics” may be given to the gut or bloodstream [14,23]. However, for some postbiotic efficacy, such as cell wall components, specific microorganisms are effective [24,25]. These properties may be important factors in designing studies on the effective effects of specific postbiotics on metabolic disorders.

### 3.1. SCFAs

SCFAs are produced by intestinal microbes by digesting vegetable polysaccharides; they are typically composed of 1–6 carbon-based anions [43]. Dietary fiber, i.e., undigested dietary carbohydrates that reach the large intestine, is the most important energy source for glycolytic microorganisms in the large intestine; as microbial consumption of dietary fiber increases, so does the production of SCFAs [44].

Butyrate (C4), propionate (C3), and acetate (C2) are the most representative intestinal SCFAs [43], and they are found in the large intestine and feces at a ratio of approximately 60:20:20 [45]. SCFAs promote the growth of beneficial bacteria by converting the intestinal milieu to be more acidic [20]. In addition, they are known to have anti-inflammatory [46], appetite modulation, cardiovascular disease prevention [47], anti-obesity, and diabetes prevention properties [48,49]. Features of each SCFA type are as follows:

Butyric acid is a major source of energy in colon cells [43]. It modulates cell proliferation and differentiation, and has immunomodulatory and anti-inflammatory properties [49,50]. In addition, butyric acid may control gene expression by inhibiting histone deacetylases [51]. Butyric acid is one of the most important energy sources in intestinal cells and has long been used to treat colon disorders. When butyric acid is supplied to the colon of patients with colorectal cancer, inflammation is reduced compared to that in individuals who received a placebo [50].

Propionic acid is one of the primary substrates of gluconeogenesis, the process by which glucose is produced in the liver [52]. By slowing energy absorption in humans, propionic acid prevents long-term weight gain [48] and improves animals’ physical ability [53]. Gao et al. [52] confirm that propionic acid protects hepatocytes from palmitic acid-induced lipotoxicity in calves. Lipotoxicity is a precursor of nonalcoholic steatohepatitis and destroys hepatocytes while activating hepatic stellate and immune cells [54].

Although acetic acid is less abundant than butyric acid and propionic acid, it has anti-inflammatory and analgesic properties [55]. In addition to increasing energy expenditure and releasing intestinal hormones, acetic acid may help manage hunger and weight in the central nervous system, implying that it may help prevent cardiovascular disease [47]. When an acetic acid-rich diet is consumed, resistance to enterohemorrhagic Escherichia coli (O157: H7) is boosted [56], probably because acetic acid adheres to the intestinal barrier and inhibits toxins from entering the circulatory system.

### 3.2. Extracellular Polymeric Substances (EPSs)

Bacterial polysaccharides are produced both intra- and extra-cellularly. EPSs are composed of a capsule that is covalently bound to the cell surface and a slime layer that is loosely attached to the cell surface or secreted to the outside [57]. EPSs are diverse; thus, they may be classified using various criteria. The most common criterion is to divide them into homopolysaccharides, composed of the same polysaccharide, and heteropolysaccharides, composed of two or more polysaccharide types [58]. Bacterial EPSs have different physical properties depending on the type and structural characteristics of the constituent sugars, and are easily degraded and recovered [59]. Therefore, EPSs are used in various industrial fields, e.g., as coagulants, emulsifiers, and stabilizers. Because they have a function similar to that of lactic acid bacteria, they have attracted attention for medical usage [60].

### 3.3. Enzymes

Enzymes are active proteins found in all living organisms that catalyze various biological reactions [61]. Microorganism enzymes break down components such as carbohydrates, proteins, and fats into individual monomers to aid in human digestion and bioavailability, offer flavor and texture to food, and convert unsaturated fatty acids to saturated fatty acids [62,63]. When probiotics are added to the diet of farmed fish, digestive enzymes generated by the probiotics increase digestion and absorption of the fish feed, thereby increasing growth [64]. Therefore, industrial enzymes have been produced by a variety of microorganisms, ranging from eukaryotes such as yeast and molds to prokaryotes, comprising both gram-positive and gram-negative bacteria [61]. Lactic acid bacteria have been extensively investigated because they produce lactases, proteases, peptidases, fructanases, amylases, bile salt hydrolases, phytases, and esterases [63]. For numerous years, enzymes produced by lactic acid bacteria have been used in commercial foods and beverages, improving the safety of fermented products by producing various compounds that inhibit harmful bacteria [65]. However, studies of the utilization of antioxidant enzymes in vivo are limited [66].

### 3.4. Vitamins

Vitamins are essential nutrients required for metabolic activity in living cells [67]. Because humans are unable to synthesize most vitamins [68], they must be obtained through the diet, mainly from dairy- and grain-based foods, fruits, and vegetables. Some vitamins, such as vitamins K and B, are synthesized by gut bacteria and probiotics [22]. Dietary B vitamins are absorbed in the small intestine, whereas B vitamins produced by microorganisms are absorbed by the large intestine [69], suggesting the possibility that B vitamins derived from diet and intestinal microbes are metabolized differently in the human body. Water-soluble B vitamins such as biotin, cobalamin, folic acid, nicotinic acid, pantothenic acid, pyridoxine, riboflavin, and thiamine are synthesized by human intestinal microbiota [22]. Cobalamin is a vitamin that is only synthesized by anaerobic bacteria and not by animals, plants, or fungi [67].

The vitamins most commonly synthesized by human gut microbiota are riboflavin and niacin [69]. Riboflavin is a nutrient required for healthy maintenance of the human nervous, endocrine, cardiovascular, and immune systems [70]. According to Aljaadi et al. [71], riboflavin intake below the average requirement may increase the risk of anemia. B vitamins are important cofactors and coenzymes in many metabolic pathways and play a significant role in immunological homeostasis [68]. Dietary components and gut microbiota modulate host immune function via B vitamins. When compared to chemically manufactured vitamins, vitamins synthesized using gut bacteria are more consumer-friendly and less prone to induce side effects [67].

### 3.5. Cell Wall Components

Probiotic cell wall components have beneficial effects on human health; they may be used for therapeutic purposes. Peptidoglycan and teichoic acid, the major components of the cell wall, have immunomodulatory effects [31,33]. Teichoic acid is classified into two types: lipoteichoic acid and wall teichoic acid. Lipoteichoic acid binds to the bacterial membrane through a glycolipid, whereas wall teichoic acid forms covalent bonds with peptidoglycans [72]. Various Lactobacillus-derived peptidoglycans inhibit the release of inflammatory cytokines in an LPS-stimulated RAW 264.7 cell model [24,33]. In addition, peptidoglycan derived from L. rhamnosus improves the innate immune response in Streptococcus-infected immunodeficient mice [31]. Lipoteichoic acid may also be effective in treating skin infections. Zong et al. [73] find that lipoteichoic acid-producing gut microbes play an important role in preventing bacterial and viral infections by inducing the release of antimicrobial peptides such as β-defensin and cathelicidin. In contrast, Tominari et al. [25] have reported that lipoteichoic acid does not reduce inflammation, but instead induces inflammatory alveolar bone loss. Therefore, more research on the anti-inflammatory effects of lipoteichoic acid is required.

### 3.6. Cell-Free Supernatants of Bacteria

Bacterial cell-free supernatants are obtained by centrifugation of cell cultures to collect biologically active metabolites such as SCFA, EPSs, enzymes, and vitamins [22]. Similar to bacteria, active metabolites released by bacteria have beneficial effects on human health. According to Kuley et al. [74], bacterial culture supernatants show higher antioxidant capacity than intact cells. Culture supernatants from different bacteria have several functions. For example, supernatants from Lactobacillus paracasei cultures suppress obesity in rats [35], whereas those from L. rhamnosus GG inhibit alcoholic liver disease in mice [36] and those from L. casei DG reduce intestinal inflammation in an ex vivo organ culture model [37].

## 4. The Role of Postbiotics in Different Metabolic Disorders

Altering factors of gut microbiota such as diet, exercise, and drugs can result in alterations to gut microbiome that influence host metabolism. Gut dysbiosis can modify the host’s ability to utilize energy from ingestions, as well as progress to metabolic diseases. Thus, postbiotics have potentials to alleviate metabolic disease factors without the need to alter the gut microbiota.

### 4.1. Regulation of Low-Grade Inflammation

Inflammation is a bioprotective reaction that restores body function in response to a variety of stimuli, such as damage caused by external factors and bacterial infections [75]. Acute and chronic inflammation are the two types of inflammatory responses; acute inflammation is an immunological response triggered either by a pathogen, injury, or trauma; it is a short-term high-grade inflammation response [76,77]. In contrast, chronic inflammation is caused by multiple factors, such as poor diet, stress, lack of physical activity, and environmental pollution that may lead to serious inflammatory disorders and cancer [75]. LPS, a component of the cell wall of gram-negative bacteria, causes inflammation in eukaryotic hosts. LPS also induces the production of inflammatory mediators, such as nitric oxide and proinflammatory cytokines, including tumor necrosis factor (TNF)-α, interleukin (IL)-1, and IL-6, and inhibits the production of anti-inflammatory cytokines, such as IL-10 [78]. Overproduction of inflammatory cytokines results in acute and chronic inflammation, which may lead to inflammatory disorders.

Probiotics and postbiotics are effective anti-inflammatory agents. SCFAs, as postbiotics, control the immune system through various mechanisms, including the activation of G protein-coupled receptors (GPCRs) and inhibition of histone deacetylase [79]. SCFAs inhibit TNF-α, IL-6, and nitric oxide production in macrophages and enhance the release of IL-10 [80], but inhibit IL-10 in mononuclear cells [81]. Thus, SCFAs may have varied effects on different cell types. The EPSs of Lactobacillus sp. extracted from healthy human vaginas lower TNF-α production in cervical cancer HeLa cells and show significant anti-inflammatory effects by increasing the production of IL-10 [29]. In addition, peptidoglycan, a major component of the cell wall, inhibits the release of proinflammatory cytokines such as TNF-α, IL-1β, and IL-6, which occur when macrophages are stimulated with LPSs [24].

### 4.2. Anti-Obesity Effects

Obesity is a major health issue worldwide; research on managing obesity through energy consumption has recently received attention [82]. Obesity is caused by an imbalance between energy intake and consumption, which negatively impacts various metabolic pathways in the body [5]. Although diet is an important factor in host metabolism, energy homeostasis malregulation due to excessive food intake leads to obesity [8]. High-calorie diets such as high-fat and high-sugar diets are considered the greatest risk factors for obesity [83].

SCFAs activate GPCR43, inhibit excessive energy and insulin-mediated fat accumulation by white adipose tissue, and regulate energy balance by promoting fat consumption [84]. GPCR43 overstimulation may be a treatment method for metabolic disorders, including obesity and diabetes [85]. SCFAs stimulate peptide YY (PYY) and glucagon-like peptide-1 (GLP-1) secretion from L cells that secrete hormones in the intestine [84]. PYY and GLP-1 are hormones that suppress appetite: they are released from the intestine and function as signals that give the brain a feeling of satiety [86]. According to van der Beek et al. [87], when acetic acid is injected into the distal colon of obese volunteers, fat oxidation is promoted, and the concentration of PYY increases compared to that in individuals who received a placebo. Propionic acid also stimulates PYY and GLP-1 secretion in human colon cells [48]. These results suggest that SCFAs effectively prevent and suppress obesity by participating in various human metabolic processes.

### 4.3. Antidiabetic Effects

Diabetes mellitus is a chronic disease characterized by elevated blood sugar levels [88]. Diabetes is divided into two types: type 1 diabetes (T1D) and type 2 diabetes (T2D). T1D is caused by autoimmune-cell destruction and no insulin secretion, whereas T2D is caused by insulin resistance, where insulin is secreted, but the pancreas loses function, and peripheral tissues and organs such as the liver, muscles, and adipose tissue have a reduced ability to respond [89]. Diabetes prevalence is steadily increasing worldwide, and its causes are very diverse due to genetic factors, unhealthy eating habits, lack of exercise, and obesity. An imbalance in intestinal microbiota may be a fundamental cause of diabetes [88].

Probiotics and postbiotics have antidiabetic properties [41]. SCFAs bind to GPCR41 and GPCR43 at the end of the intestine to produce the intestinal hormones PYY and GLP-1, affecting satiety and glucose homeostasis [85]. Propionic and butyric acids improve glucose and energy homeostasis by inducing intestinal gluconeogenesis and sympathetic nerve activity [90]. When butyric acid is administered orally for 4 weeks, peripheral insulin sensitivity in healthy patients significantly improves, but glucose metabolism in patients with metabolic syndrome is not affected [91]. Therefore, butyric acid has no potential as a therapeutic agent for glucose control in patients with T2D.

Insulin absorbs glucose from the human body; when insulin is insufficient, glucose cannot be used as an energy source and remains in the blood, maintaining high blood sugar levels [90]. Glucose in the blood accumulates as fat; the improvement of insulin sensitivity is very important [85]. Oral administration of the EPSs producing Bifidobacterium animalis IPLA R1 to rats fed a high-fat diet decreases serum insulin content without significant fluctuations in fasting glucose and homeostasis model assessment (HOMA) indices [92]. Therefore, EPSs have a positive effect on insulin sensitivity.

### 4.4. Prevention of Hypertension

Hypertension is a major cause of cardiovascular diseases such as myocardial infarction and strokes [6]. Preventing hypertension may reduce the risk of cardiovascular disease. A high-fat diet induces changes in the gut microbiota, disrupts the intestinal barrier, and increases plasma LPS levels, inducing oxidative stress [93]. According to a study by Li, Zhao, Wang, Chen, Tao, Tian, Wu, Liu, Cui, Geng, Zhang, Weldon, Auguste, Yang, Liu, Chen, Yang, Zhu, and Cai [6], patients with hypertension have fewer essential bacteria required for healthy conditions, such as Faecalibacterium, Oscillibacter, Roseburia, Bifidobacterium, Coprococcus, and Butyrivibrio, than those of healthy controls. Moreover, reduced nicotinamide adenine dinucleotide phosphate oxidase, a major source of free radicals in blood vessels, may be involved in hypertension and intestinal barrier dysfunction [94].

SCFAs influence blood pressure in humans through various mechanisms. Butyric acid diminishes angiotensin II-induced hypertension in mice [95], and some SCFAs are known to have ligands that can potentially be engineered as therapeutic targets for hypertension (e.g., GPR41, GPR43, GPR109a in mice and SCFA receptors such as Olf78/OR51E2 in human) [96]. Thus, supplementation with SCFAs may have a positive effect on the prevention of cardiovascular diseases.

### 4.5. Effects on Cholesterol Levels

Cholesterol accumulation is one of the leading causes of cardiovascular disease, resulting in a high number of deaths [97]. Therefore, lowering blood cholesterol levels is critical for preventing cardiovascular disease. HDL cholesterol removes cholesterol from the bloodstream and prevents arteriosclerosis, while low-density lipoprotein (LDL) cholesterol causes cholesterol to accumulate in blood vessels [98]. A significant correlation exists between high HDL cholesterol and low coronary heart disease risk, whereas low HDL cholesterol levels are correlated with early cardiovascular disease in younger patients [99].

Probiotics and postbiotics reduce cholesterol levels in the body through direct or indirect mechanisms. In an experiment where Bifidobacterium longum SPM1207 is disrupted by sonication, and postbiotics are isolated and used to analyze their effect on high-cholesterol fed rats [26], compared to the controls, serum total cholesterol and LDL cholesterol levels are significantly reduced, and HDL cholesterol levels are slightly increased in high-cholesterol postbiotic-fed rats. In recent years, dietary supplementation with probiotic metabolites such as EPSs has gained popularity to lower blood cholesterol levels. Bhat and Bajaj [30] have observed that the EPSs of Lactobacillus paracasei M7 have high cholesterol-lowering activity (70.78%) in vitro. Thus, the intake of probiotics may help manage mild hypercholesterolemia. However, further studies are needed to investigate the exact mechanism of this cholesterol-lowering action.

## 5. Conclusions

Postbiotics are metabolites or cell wall components secreted by live bacteria or released after bacterial lysis; these substances confer host health benefits. Postbiotics produced by intestinal microbiota are closely related to host conditions and the intestinal environment. As research to elucidate the functional mechanism of postbiotics continues, the regulatory function of individual postbiotics will be elucidated. Furthermore, postbiotics are promising materials that overcome the constraints of probiotic stability and safety because they are in a dead cell state, have low reactivity, are easy to process (for example, by heat treatment), and are suitable for storage and transportation.

To date, research has focused on demonstrating correlations or elucidating the functionality of specific microbes, such as probiotics. Isolation and identification of intestinal microbiota and their metabolites remain difficult, requiring specialized technology and more complex methods than normal bacterial culture conditions; therefore, materials that may be exploited as postbiotics are currently limited. To compensate for postbiotic limitations, it will be necessary to establish a diet that uses postbiotics to control the microbiome and study the efficacy of the human body. Thus, effective postbiotic materials have great potential for application in next-generation therapies for metabolic and immunological disorders, going beyond the health function improvement effects of existing probiotics and prebiotics. It is envisaged that this will revitalize the food market and energize the development of the national food industry.

## Figures and Tables

**Table 1:** In vitro and in vivo studies that highlight physiological effects by postbiotics.

Part	Bacteria	Derived Postbioics	Type of Study	Physiological Effects	References
*Bifidobacterium*	*Bifidobacterium longum* SPM1207	Dead cell (sonication)	In vivo: Male rats	Reduce total and LDL cholesterol	Shin et al. [26]
	*Bifidobacterium longum* CECT-7347	Dead cell (heat killed)	In vivo: *Caenorhabditis elegans* In vitro: Human colonic epithelial cells (HT-29)	Anti-inflammatory (Reduce acute inflammatory response, gut-barrier disruption)	Martorell et al. [2]
	*Bifidobacterium animalis subsp. lactis CECT 8145*	Lipoteichoic acid	In vivo: *Caenorhabditis elegans*	Anti-obesity (Reduce nematode’s fat content)	Balaguer et al. [27]
	*Bifidobacterium longum* W11	Exo-polysaccharide (EPS)	In vitro: Human lung fibroblasts (MRC-5)	Antioxidant (Reduce ROS, Increase RSH levels of H_2_O_2_ treated MRC-5)	Inturri et al. [28]
	*Bifidobacterium longum* W11	EPS	In vitro: Human peripheral blood mononuclear cells (PBMCs)	Immunomodulation (Immune stimulatory in ConA-stimulated PBMCs)	Inturri et al. [21]
	*Bifidobacterium longum* 35624	EPS	In vitro: Human peripheral blood mononuclear cells (PBMCs). Human monocyte-derived dendritic cells (MDDCs)	Anti-inflammatory (Modulate cytokine secretion and NF-κB activation)	Schiavi et al. [3]
*Lactobacillus*	*Lactobacillus sp.*	EPS	In vitro: Human Cervical Carcinoma cells (HeLa)	Anti-inflammatory (Decrease TNF-α, Increase IL-10)	Sungur et al. [29]
	*Lactobacillus paracasei* M7	EPS	In vitro	Reduce total cholesterol	Bhat and Bajaj [30]
	*Lactobacillus rhamnosus* CRL1505	Peptidoglycan	In vivo: Male Swiss-albino mice	Immunomodulation (Improve innate immune response, Respiratory and systemic adaptive immune response)	Kolling et al. [31]
	*Lactobacillus plantarum*	Lipoteichoic acid	In vitro: Human monocyte-like cells (THP-1)	Immunomodulation (Attenuate the pro-inflammatory signaling in THP-1 cells)	Kim et al. [32]
	*Lactobacillus acidophilus*, *Lactobacillus reuteri*, *Lactobacillus plantarum*	Lipoteichoic acid	In vitro: RAW264.7 cells	Immunomodulation (Inhibit LPS-induced TNF-α production)	Matsuguchi et al. [33]
	*Lactobacillus paracasei* D3–5	Lipoteichoic acid	In vivo: Male C57BL/6J mice	Anti-inflammatory (Enhance mucin (Muc2) expression by modulating TLR-2/p38-MAPK/NF-kB pathway)	Wang et al. [34]
	*Lactobacillus paracasei*	Bacterial lysates	In vivo: Wistar albino male rats	Anti-obesity (Reduce total serum lipids and serum triglyceride)	Osman et al. [35]
	*Lactobacillus rhamnosus* GG	Bacterial lysates	In vivo: Male mice	Preventing alcoholic liver disease	Wang et al. [36]
	*Lactobacillus casei* DG	Bacterial lysates	Ex-vivo: Organ culture model	Immunomodulation (Relieve inflammatory response of the intestinal mucosa)	Compare et al. [37]
	*Lactobacillus rhamnosus* GG	Bacterial lysates: Protein (HM0539)	In vitro: Intestinal epithelial cells (Caco-2) In vivo: C57BL/6 mice	Protect intestinal epithelium Prevent colitis, intestinal barrier dysfunction, bacteria translocation	Gao et al. [38]
	*Lactobacillus amylovorus* CP1563	Dead cell (heat killed)	In vivo: Male C57BL/6N mice	Anti-obesity (Prevention and treatment of dyslipidemia)	Nakamura et al. [39]
*Bacillus*	*Bacillus subtilis sp.*	Polysaccharide	In vivo: Male Sprague–Dawley rats	Decrease glucose levels in the blood Increase insulin levels in the serum	Ghoneim et al. [40]
	*Bacillus licheniformis*	Polysaccharide	In vivo: Male Wistar rats	Prevent diabetes complications (Increase glycogen level, SOD, CAT Decrease glucose level, TBARS, hepatic, and renal indices’ toxicity)	Dahech et al. [41]
	*Bacillus velezensis Kh2–2*	Bacterial lysates	In vitro: RAW264.7 cells In vivo: Male mice Ex-vivo: Splenocytes in mouse	Immunomodulation (Induce NO, immune-related cytokine in RAW264 Induce IL-2, IFN-γ and inhibit IL-10 in the ex vivo study Enhance innate and adaptive immunity, stimulate immune-related cytokine secretion, and modulate gut microbiota dysbiosis in mice)	Mi et al. [42]
